# vIL-10-overexpressing human MSCs modulate naïve and activated T lymphocytes following induction of collagenase-induced osteoarthritis

**DOI:** 10.1186/s13287-016-0331-2

**Published:** 2016-05-18

**Authors:** Eric Farrell, Niamh Fahy, Aideen E Ryan, Cathal O Flatharta, Lisa O’Flynn, Thomas Ritter, J Mary Murphy

**Affiliations:** Department of Oral and Maxillofacial Surgery, Special Dental Care and Orthodontics, Erasmus MC, University Medical Centre, Room Ee1614, Erasmus MC, Wytemaweg 80, Rotterdam, 3015CN The Netherlands; Regenerative Medicine Institute, National University of Ireland Galway, Galway, Ireland; Musculoskeletal Regeneration, AO Research Institute Davos (ARI), Davos, Switzerland; College of Medicine, Nursing and Health Sciences, National University of Ireland Galway, Galway, Ireland; Orbsen Therapeutics Ltd, Galway, Ireland; Discipline of Pharmacology and Therapeutics, National University of Ireland Galway, Galway, Ireland

**Keywords:** Collagenase-induced osteoarthritis, Mesenchymal stem cell, vIL-10, Cell therapy, Gene therapy, Xenogeneic

## Abstract

**Background:**

Recent efforts in osteoarthritis (OA) research have highlighted synovial inflammation and involvement of immune cells in disease onset and progression. We sought to establish the in-vivo immune response in collagenase-induced OA and investigate the ability of human mesenchymal stem cells (hMSCs) overexpressing viral interleukin 10 (vIL-10) to modulate immune populations and delay/prevent disease progression.

**Methods:**

Eight-week-old male C57BL/6 mice were injected with 1 U type VII collagenase over two consecutive days. At day 7, 20,000 hMSCs overexpressing vIL-10 were injected into the affected knee. Control groups comprised of vehicle, 20,000 untransduced or adNull-transduced MSCs or virus alone. Six weeks later knees were harvested for histological analysis and popliteal and inguinal lymph nodes for flow cytometric analysis.

**Results:**

At this time there was no significant difference in knee OA scores between any of the groups. A trend toward more damage in animals treated with hMSCs was observed. Interestingly there was a significant reduction in the amount of activated CD4 and CD8 T cells in the vIL-10-expressing hMSC group.

**Conclusions:**

vIL-10-overexpressing hMSCs can induce long-term reduction in activated T cells in draining lymph nodes of mice with collagenase-induced OA. This could lead to reduced OA severity or disease progression over the long term.

**Electronic supplementary material:**

The online version of this article (doi:10.1186/s13287-016-0331-2) contains supplementary material, which is available to authorized users.

## Background

Although osteoarthritis (OA) is typically characterised by loss or damage to articular cartilage, inflammation of the synovial membrane is a prevalent feature believed to contribute to both symptoms and disease progression. Thickening of the synovial membrane has been identified in patients with early-stage OA, and increased vascular density and cellular infiltration of the synovium is a prominent feature of disease pathogenesis [1]. Macrophages localised to the synovial lining are primary mediators of inflammation in the joint, responsible for the production of pro-inflammatory cytokines such as tumour necrosis factor alpha (TNF-α) and interleukin (IL)-1β, which can induce destructive processes in neighbouring cartilage [2, 3]. T lymphocytes are the most abundant infiltrating immune cells present in OA synovium, with both CD4^+^ effector T cells and CD8^+^ cytotoxic T cells observed in the sublining layer [4, 5]. Moreover, a role of CD4^+^ T cells in the pathogenesis of OA has been identified, through the induction of MIP-1γ and osteoclastogenesis [6].

Mesenchymal stem cells (MSCs) have been considered a promising cell source for the repair of damaged cartilage in OA, due to their chondrogenic differential potential [7]. In addition to their multipotent nature, MSCs may enhance intrinsic tissue repair through the release of trophic factors which act to modulate inflammatory processes or recruit endogenous progenitor cells [8, 9]. MSCs have the ability to polarise pro-inflammatory macrophages towards an anti-inflammatory phenotype and suppress T-cell proliferation, and have been previously reported to exert anti-inflammatory effects on human osteoarthritic synovium in vitro [10–12]. Xenotransplantation of human MSCs (hMSCs) has enhanced meniscal regeneration and prevented OA progression in rats following hemi-meniscectomy, with no significant difference observed between the reparative effects of xenogeneic or rat syngeneic MSCs [13]. Furthermore, allogeneic MSCs have been shown to prevent post-traumatic arthritis following intra-articular fracture in mice; however, an effect of hMSC delivery on synovial hyperplasia was not observed [14]. Although intra-articularly delivered adipose-derived stem cells have been reported to migrate to the synovium, reduce synovial lining thickness and decrease cartilage damage in a murine collagenase-induced OA model [15], the ability of bone marrow-derived hMSCs to alter the inflammatory environment in OA and subsequently delay disease progression requires further investigation.

One factor of particular interest to target OA-associated inflammatory processes is IL-10. IL-10 is a 34 kDa homodimeric cytokine produced by activated macrophages, T-helper type 2 cells and B cells, and is associated with diverse biological responses [12, 16–18]. IL-10 can suppress pro-inflammatory cytokine production; for example, TNF-α, IL-1β and IL-6 produced by activated macrophages, monocytes and T-helper type 1 cells respectively [17, 19]. Furthermore, IL-10 reduces monocyte expression of major histocompatibility complex (MHC) class II, resulting in decreased antigen presentation and subsequent inhibition of T-cell proliferation [20]. In addition to its immunosuppressive properties, IL-10 can stimulate B cells to increase MHC class II expression and immunoglobulin production, as well as enhance mast cell growth [21–23]. Human IL-10 exhibits 84 % amino acid sequence homology to an open reading frame product of Epstein–Barr virus, termed viral IL-10 (vIL-10) [24]. Although vIL-10 displays comparable immunosuppressive activity to human IL-10, it lacks particular stimulatory functions [22]. Furthermore, adenoviral-mediated gene transfer of vIL-10 or overexpression by retrovirally transduced MSCs has been reported to significantly decrease the frequency of arthritis, delay the onset and reduce the severity of arthritic symptoms in a murine collagen-induced rheumatoid arthritis model [25, 26].

In the present study, we have investigated the ability of xenogeneic hMSCs overexpressing vIL-10 to modulate the inflammatory environment and alter disease progression in a murine collagenase-induced OA model. Overexpression was induced using an adenoviral construct with transduced hMSCs referred to as AdIL-10 MSCs. This model is associated with joint ligament damage resulting in instability [27], as well as synovial activation compared with other instability-related OA models [28]. Intra-articular injection of 1 U collagenase was administered over two consecutive days to induce mild structural changes and OA progression in order to achieve a suitable model to evaluate the potential of an anti-inflammatory component to attenuate OA pathogenesis. Our findings highlight modulatory activity of hMSCs adenovirally transduced to overexpress vIL-10 on the levels of naive and activated CD4^+^ and CD8^+^ T cells in vivo during OA progression.

## Methods

### Isolation and culture of hMSCs

hMSCs were obtained from bone marrow aspirates taken from the iliac crest of healthy human donors. All procedures were performed with informed consent and approved by the Clinical Research Ethical Committee at University College Hospital, Galway, Ireland. The cells were isolated based on plastic adherence as described previously [29] and expanded in alpha-Minimum Essential Medium (α-MEM; Life Technologies, Zoetermeer, the Netherlands) containing 10 % pre-screened foetal bovine serum (FBS; Hyclone, South Logan, UT, USA), 1 % penicillin/streptomycin and 1 ng/ml recombinant human basic fibroblast growth factor (FGF2; PeproTech, London, UK). Upon reaching confluency, hMSCs were subcultured to passage 3 for adenoviral transduction. Three MSC donors were used for the in-vitro experiments and one of these was used for the in-vivo study with eight replicates per condition.

### Adenoviral transduction of hMSCs

Adenoviral transduction of hMSCs was performed using the lanthanide-based method as described previously [30]. hMSCs were seeded at a density of 6 × 10^3^ cells/cm^2^ in a T175 flask 24 h prior to transduction. Calculated amounts of adenoviral vectors expressing vIL-10 (AdIL-10) or Null virus (AdNull) were added to serum-free α-MEM medium to generate a final multiplicity of infection of 100. LaCl_3_ (Sigma-Aldrich, Wicklow, Ireland) was dissolved in deionised water to generate a 0.4 M stock solution and stored at 4 °C. A working solution of 0.04 mM LaCl_3_ was generated following the addition of an appropriate volume of a 0.4 M stock to serum-free medium. An equal volume of 0.04 mM LaCl_3_ solution was added to the virus solution, mixed gently and incubated at room temperature for 30 minutes. hMSCs were incubated with the LaCl_3_/virus mixture for 3 h, following which cells were washed twice with serum-containing medium. Cells were harvested for subsequent experiments 48 h post transduction.

### In-vitro assessment of immunomodulatory properties of AdIL-10 MSC CM

Macrophages were seeded at a density of 4 × 10^5^ cells/ml in a 24-well plate, in RAW264.7 culture medium (Dulbecco’s modified Eagle’s medium (4500 ng/ml glucose), 2 mM glutamine, 10 % FBS and 1 % penicillin/streptomycin). Lipopolysaccharide (LPS; Sigma-Aldrich) was added to the cell culture medium at a final concentration of 0.5 ng/ml. Supernatant was harvested at 2, 4, 6, 12 and 24 h post LPS stimulation and centrifuged at 400 × *g* for 5 minutes to pellet any debris. TNF-α levels in the supernatant were measured with a commercial human TNF-α DuoSet ELISA kit according to the manufacturer’s instructions (R&D Systems, Minneapolis, Minnesota). To assess the effect of MSC conditioned medium (CM) (*n* = 3 donors) on TNF-α production by LPS-activated mouse macrophages, the vIL-10 concentration was quantified in the CM from AdIL-10-transduced MSCs by ELISA (purified rat anti-human vIL-10 (capture antibody); BD Biosciences; and biotinylated rat anti-human vIL-10 (detection antibody); BD Biosciences, Oxford England) diluted to ensure addition of 100 ng/ml vIL-10 per well. Equal volumes (compared with AdIL-10 MSC CM) of untransduced MSC CM and AdNull-transduced MSC CM were used as controls.

### T-cell proliferation assays and mixed lymphocyte reaction cultures

Lymphocytes were obtained from the lymph nodes and spleens of C57BL/6 mice. Lymphocytes were washed with 0.1 % BSA/PBS and stained in pre-warmed (37 °C) 10 μM Vybrant CFDA SE (carboxy-fluorescein diacetate, succinmidyl ester (CFSE))/PBS staining solution (Invitrogen, Carlsbad, CA, USA) as per the manufacturer’s instructions. Then 1 × 10^5^ CFSE-stained T cells were stimulated with phorbol myristate acetate (5 ng/ml) and ionomycin (400 ng/ml) in T-cell medium (RPMI 1640 supplemented with 10 % FCS, 50 μM β-mercaptoethanol, 100 U/ml penicillin, 0.1 mg/ml streptomycin, 1 mM sodium pyruvate and 2 mM l-glutamine). Lymphocytes were co-cultured with human untransduced MSCs, AdNull MSCs and AdIL-10 MSCs (*n* = 3 donors) at MSC:T-cell ratios ranging from 1:400 to 1:10 in a humidified incubator for 4 days. Mouse T-cell proliferation (CFSE quantification) was measured by flow cytometry (FACS Canto; BD Biosciences).

For mouse mixed lymphocyte reactions, human untransduced MSCs, AdNull MSCs and AdIL-10 MSCs (*n* = 3 donors) were plated in 96-well round-bottom plates. CFSE-labelled untreated lymphocytes isolated from C57BL/6 mice were used as responders, at ratios of 1:5, 1:10 and 1:50 MSC:T cells. A total of 4 × 10^3^, 2 × 10^4^ or 4 × 10^4^ stimulating cells were co-cultured with 2 × 10^5^ CFSE-labelled responding lymphocytes for 5 days. T-cell proliferation was analysed on a FACS Canto (BD Biosciences).

### OA model

All procedures were approved by the Animal Care and Research Ethics Committee of the National University of Ireland, Galway and conducted under licence issued by the Department of Health & Children, Ireland. Eight-week-old male C57BL/6 mice were supplied by Charles River Laboratories, Ballina, Ireland, UK. All animals were kept on a 12 h light/dark cycle with ad libitum access to water and standard laboratory chow. Eight animals were randomly assigned to each treatment group with the treatment leg (left or right) also chosen randomly. All animals were coded and scorers were blinded to the treatment.

To induce OA, 1 U of highly purified bacterial type VII collagenase (Sigma-Aldrich) in 6 μl of physiological saline (vehicle) was injected into the knee joint of mice twice over two consecutive days (totalling 2 U). Animals were anaesthetised using isoflurane and given a subcutaneous injection of buprenorphine (Temgesic, 0.01 mg/kg body weight) prior to collagenase injection. One week later animals were injected with one of five conditions in a volume of 6 μl: vehicle alone, 2 × 10^4^ MSCs, AdIL-10-transduced MSCs, AdNull-transduced MSCs or the equivalent amount of virus used to transduce 2 × 10^4^ cells suspended in the vehicle. The cell number was chosen based on ELISA-based analysis of the amount of vIL-10 produced by the hMSCs and corresponded to 100 ng/ml vIL-10 production. Seven weeks after the induction of OA, animals were euthanised and the joints were removed, fixed in 10 % formalin and subsequently decalcified in 10 % EDTA. Popliteal and inguinal lymph nodes were also harvested and analysed for T-cell markers by flow cytometry. A timeline of the model is provided in Additional file 1: Figure S1.

### Histological scoring of sections

Paraffin sections (5 μm) were stained with safranin O and fast green. Sections were deparaffinised by immersion of slides twice in 100 % xylene for 5 minutes, and rehydrated to distilled water following immersion in 100 % ethanol for 2 minutes and for 1 minute in 95 % and 70 % ethanol. Sections were stained utilising 0.02 % fast green for 4 minutes, acetic acid for 3 seconds and 0.1 % safranin O for 6 minutes. Following dehydration, sections were mounted with DPX (all Sigma-Aldrich). To score the femurs and tibiae, six sections per animal, spaced approximately 100 μm apart, were scored by three independent, blinded observers. The scoring was based upon the semi-quantitative grading system designed by the OARSI working group [31] with damage ranging from 0 (no damage) to 6 (complete denudation of the cartilage). All scores for each knee compartment (lateral and medial tibia and femur) were added separately and averaged and were then averaged for the three observers. To score the amount of synovial inflammation, a grading of 0–3 was used, with 0 indicating normal synovium with one or two layers of cells and 3 being several cell layers thick. Grades 1 and 2 fell in between these two categories. Finally a total knee damage score was generated by summing all scores for the six compartments for each knee. Representative images of safranin O-stained slides are available in Additional file 2: Figure S2.

### Thionine staining

Paraffin sections (5 μm) were de-waxed and redehydrated in xylene and decreasing concentrations of alcohol (100 %, 96 % and 70 % twice each for 5 minutes respectively). Samples were then stained in 0.04 % thionine in 0.01 M aqueous sodium acetate pH 4.5 for 5 minutes. They were then differentiated in 70 % ethanol for 10 seconds, rinsed in 96 % ethanol and then taken through 100 % ethanol and xylene. Slides were mounted with Entellan.

### Multiplex ELISA

To assess monocyte chemoattractant protein-1 (MCP-1) levels in serum samples harvested at 7 weeks post induction of OA, a commercially available chemiluminescent array was utilised (16-plex mouse cytokine screen; Quansys Biosciences, Logan, Utah, US). Mice were randomised and serum from two animals per treatment group was pooled and stored at −80 °C until use. Briefly, an 8-point standard curve was prepared using antigen standard provided, and serum samples were diluted 1:2 in sample diluent. Then 50 μl of sample or standard was added to each well of a pre-antibody-coated 96-well plate. The plate was covered with a plate seal, and placed on a plate shaker at 500 RPM for 1 h at room temperature. Following sample incubation, the plate was washed three times with wash buffer and 50 μl per well of Detection Mix was added. The plate was covered with a new seal, and returned to the plate shaker for 1 h at 500 RPM at room temperature. Following six washes, 50 μl of 1× streptavidin–HRP was added per well, and the plate was incubated at room temperature for 15 minutes with shaking at 500 RPM. Following this incubation period, substrate A (hydrogen peroxide) and substrate B (signal enhancer) were mixed in a 1:1 ratio, and 50 μl was added per well. Imaging was performed immediately using a Fluorchem imager (Alpha Innotech, Dublin, Ireland) and the image was analysed using Q-View software, version 2.15 (Quansys Biosciences). All other cytokines in the array were below detectable levels.

### Analysis of T-cell subsets from the draining lymph nodes

Popliteal and inguinal lymph nodes were removed from C57BL/6 mice (ipsilateral side). Single cell suspensions were obtained from each lymph node from each animal as described previously [32]. Mice were randomised and lymph nodes from two animals per treatment group were combined for T-cell analysis, with the exception of one AdIL-10 virus-treated animal which appears as a singlet due to the macroscopic observation of a large haematoma and swelling above the treated knee joint (thus creating a fifth data point in this group). Cell suspensions were stained according to Table 1 for cell surface expression of CD4, CD8 and CD25 to characterise individual T-cell subsets. CD4 identifies T-helper cells while CD8 identifies cytotoxic T cells. Cell surface expression of CD25 was used as a marker of T-cell activation [33]. For assessment of myeloid cells, popliteal lymph nodes from two animals and inguinal lymph nodes from four animals were pooled, because of low cell number. Cell suspensions were also stained for expression of CD11b (Alex Fluor® 647) and Ly6C (Peridinin Chlorophyll Protein Complex (PerCP)/Cy5.5) (Table 1; Biolegend, Dublin, Ireland) as markers of myeloid cells and inflammatory monocytes, respectively [34]. Samples were analysed on a FACS Canto cytometer (BD Biosciences).

**Table 1:** Flow cytometry antibodies used as markers of interest

Antibody	Flurophore	Antibody dilution factor	Biolegend catalogue number
Anti-mouse CD8a	PE/Cy7	1:100	100721
Anti-mouse CD4	APC	1:120	100411
Anti-mouse CD25	FITC	1:60	101907
Anti-mouse/human CD11b	Alexa Fluor® 647	1:60	101220
Anti-mouse Ly6C	PerCP/Cy5.5	1:50	128011

### Statistical analysis

**Fig. 1:**
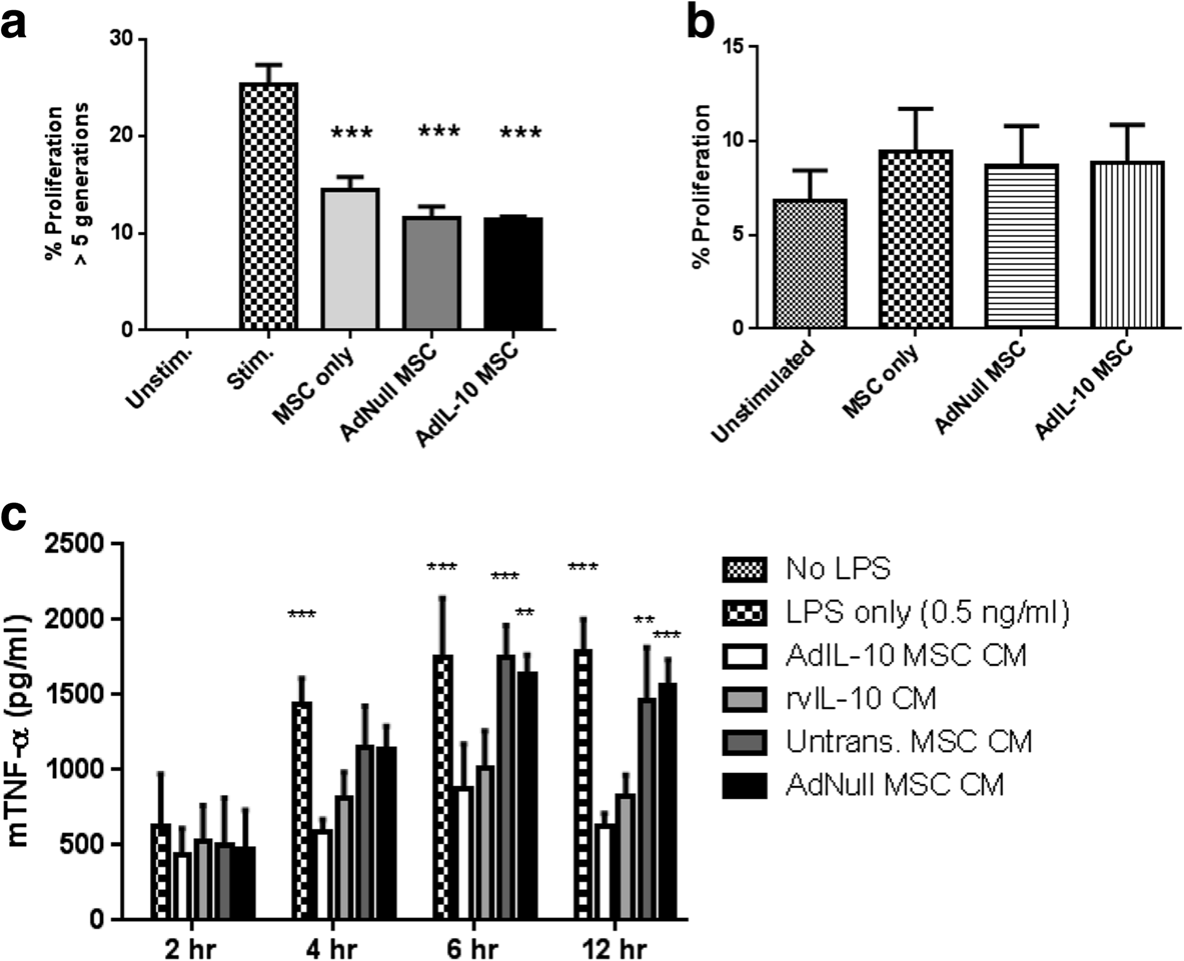
AdIL-10-transduced MSCs are immunomodulatory and not immunogenic. **a** To confirm the immunomodulatory potential of hMSCs on activated murine lymphocytes, untransduced, AdNull-transduced and AdIL-10-transduced hMSCs were co-cultured with ionomycin and PMA-stimulated C57BL/6 lymphocytes for 4 days at a ratio of 1:10. Murine lymphocyte proliferation was measured by CFSE dilution and flow cytometric detection of CFSE fluorescent peaks. Untransduced, AdNull-transduced and AdIL-10-transduced MSCs reduced the proliferation of stimulated lymphocytes, confirming their immunosuppressive capacity. Values represent the mean ± SD of three biological replicates. Statistical analysis was performed using one-way ANOVA, followed by Bonferroni’s multiple comparisons test. ***p < 0.001, indicating a significant difference compared with non-co-cultured stimulated lymphocytes. **b** To assess potential immunostimulatory activity of hMSCs, untransduced, AdNull-transduced and AdIL-10-transduced hMSCs were co-cultured with unstimulated C57BL/6 lymphocytes for 5 days, at a ratio of 1:5. Despite their xenogeneic origin, hMSCs did not induce proliferation of unstimulated C57BL/6 lymphocytes beyond basal levels. **c** To confirm immunosuppressive activity of vIL-10 overexpressed by AdIL-10-transduced MSCs, LPS-stimulated murine macrophages (RAW264.7) were treated with conditioned medium (*CM*) from untransduced, AdNull-transduced or AdIL-10-transduced hMSCs (100 ng/ml vIL-10), or 100 ng/ml recombinant vIL-10 (rvIL-10) alone. CM harvested from AdIL-10-transduced MSCs as well as rvIL-10 significantly reduced TNF-α production by LPS-activated macrophages. Values represent the mean ± SD of three biological replicates. Statistical significance was determined by a one-way ANOVA with Bonferroni’s multiple comparisons test. *p < 0.05 **p < 0.005, ***p < 0.001. *Asterisks* indicate significantly different from AdIL-10 MSC CM. *LPS* lipopolysaccharide, *MSC* mesenchymal stem cell, *TNF-α* tumour necrosis factor alpha

In-vitro data were analysed by one-way or two-way ANOVA (Fig. 1) followed by Bonferroni’s post-hoc test. Knee scores and FACS data were analysed by Kruskal–Wallis test followed by Dunn’s multiple comparisons test.

## Results

### AdIL-10-transduced hMSCs are immunomodulatory and not immunogenic towards murine lymphocytes

To confirm the immunomodulatory potential of a xenogeneic source of MSCs, the ability of untransduced, AdNull-transduced and AdIL-10-transduced hMSCs to suppress the proliferation of stimulated C57BL/6 lymphocytes in vitro was determined. Untransduced, AdNull-transduced and AdIL-10-transduced hMSCs significantly reduced the proliferation of stimulated lymphocytes (at greater than five generations) compared with untreated cells (Fig. 1a; *p* < 0.001, *n* = 3). Furthermore, AdIL-10-transduced hMSCs showed a trend towards decreased proliferation compared with untransduced cells, but this effect was not significant. At three or more generations, only AdIL-10-transduced MSCs showed a significant decrease in proliferation rate compared with stimulated but untreated splenocytes (data not shown). Given the xenogeneic source of the hMSCs we also tested the immunogenicity of these cells in culture with unstimulated splenocytes. No significant increase in splenocyte proliferation was observed with the addition of human cells whether these were MSCs, AdNull-transduced MSCs or AdIL-10-transduced MSCs (Fig. 1b; *p* = 0.9854, *n* = 3). This indicated that, at least in vitro, hMSCs were not immunogenic.

### AdIL-10-transduced hMSC CM is anti-inflammatory

To confirm that vIL-10 present in CM harvested from transduced hMSCs was anti-inflammatory, the effect on TNF-α production by LPS-stimulated RAW264.7 macrophages was determined. Macrophages were stimulated with 0.5 ng/ml LPS alone, or co-incubated with LPS and AdIL-10, AdNull or untransduced MSC CM, or medium containing an equivalent concentration of recombinant vIL-10 (100 ng/ml). At 4, 6 and 12 h post LPS stimulation there was a significant decrease in the amount of TNF-α production by AdIL-10 CM and recombinant vIL-10-treated macrophages compared with LPS only (Fig. 1c; 1.791 ± 0.21 ng/ml in LPS only vs 0.628 ± 0.08 ng/ml in AdIL-10-transduced MSC CM at 12 h, *p* < 0.001, *n* = 3). Treatment of the RAW264.7 cells with CM from untransduced or AdNull-transduced MSCs did not significantly reduce the levels of TNF-α production compared with samples treated with LPS only (1.791 ± 0.21 ng/ml in LPS only vs 1.456 ± 0.36 ng/ml in untransduced hMSC CM at 12 h).

### vIL-10-overexpressing hMSCs induce long-lasting reduction in activated T cells

To determine the immunomodulatory effect of vIL-10-overexpressing hMSCs in vivo during OA development, popliteal and inguinal lymph nodes were harvested 6 weeks post hMSC injection and analysed by flow cytometry for CD4, CD8 and the activation marker CD25. Quantification of vIL-10 release from AdIL-10-transduced MSCs in culture prior to injection showed that AdIL-10-transduced cells produced 0.858 μg/ml of AdIL-10 vs 0 ng/ml in both the untransduced and AdNull-transduced MSCs. A significant effect of injection of AdIL-10-transduced MSCs on the presence and activation state of CD4^+^ and CD8^+^ T cells was observed.

**Fig. 2:**
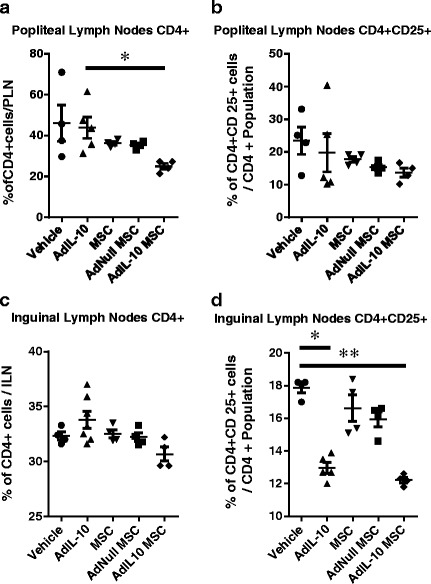
AdIL-10-transduced MSCs reduce the number of CD4^+^ T cells in the popliteal and inguinal lymph nodes 6 weeks after injection. CD4 and CD25 expression by lymphocytes isolated from the popliteal and inguinal lymph nodes, 6 weeks post treatment. **a**, **b** AdIL-10-transduced MSCs reduce CD4^+^ T-cell levels in the popliteal lymph nodes compared with AdIL-10, with no significant difference in the number of activated (CD25^+^) CD4 T cells between any experimental groups. **c**, **d** No significant difference was observed between any of the treatment groups in the amount of CD4^+^ T cells present in the inguinal lymph nodes. However, AdIL-10 only and AdIL-10-transduced MSCs significantly decreased the amount of activated CD4^+^ cells compared with vehicle-treated animals. Data points represent *n* = 4, pooled from eight animals. Data points in the AdIL-10 group represent *n* = 5, with three samples pooled from two animals and two single samples. Statistical significance was determined using a Kruskal–Wallis test, followed by Dunn’s multiple comparisons test. *p < 0.05, **p < 0.005, ***p < 0.001. *Lines* indicate significant difference between the individual groups. *ILN* inguinal lymph node, *MSC* mesenchymal stem cell, *PLN* popliteal lymph node

**Fig. 3:**
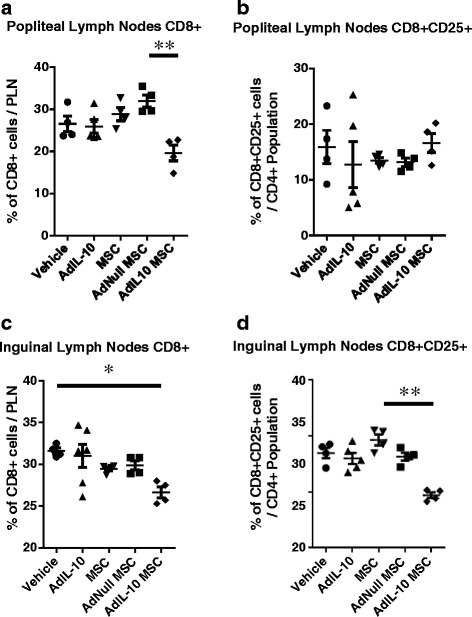
AdIL-10-transduced MSCs reduce the number of CD8 T cells in the popliteal and inguinal lymph nodes 6 weeks after injection. CD8 and CD25 expression by lymphocytes isolated from the popliteal and inguinal lymph nodes, 6 weeks post treatment. **a**, **b** AdIL-10-transduced MSCs reduced the amount of CD8^+^ T cells compared with AdNull-transduced MSCs in the popliteal lymph nodes, whereas no significant difference in the amount of activated CD8^+^ T cells was observed. **c**, **d** AdIL-10-transduced MSCs significantly decreased the amount of CD8^+^ cells in the inguinal lymph nodes and reduced the amount of activated CD8^+^ T cells compared with MSCs alone. Data points represent *n* = 4, pooled from eight animals. Data points in the AdIL-10 group represent *n* = 5, with three samples pooled from two animals and two single samples. Statistical significance was determined using a Kruskal–Wallis test, followed by Dunn’s multiple comparisons test. *p < 0.05, **p < 0.005, ***p < 0.001. *Lines* indicate significant difference between the two groups. *ILN* inguinal lymph node, *MSC* mesenchymal stem cell, *PLN* popliteal lymph node

In the popliteal lymph nodes we observed a significant reduction in the percentage of CD4^+^ T cells in the AdIL-10 MSC group compared with the AdIL-10 only group (Fig. 2a; 43.9 ± 11.6 % vs 25 ± 2.8 %; *p* = 0.0314). In the inguinal lymph nodes both the AdIL-10 virus alone and the AdIL-10-transduced MSCs had significantly lower percentages of activated CD4^+^ T cells compared with vehicle (Fig. 2d; 13 ± 0.7 % and 12.2 ± 0.3 % vs 17.9 ± 0.6 % respectively; *p* = 0.0021) (*n* = 4 samples pooled from eight animals). There was also a significant reduction in the numbers of CD8^+^ T cells in the popliteal (Fig. 3a; 32 ± 2.9 % in AdNull-transduced MSCs vs 19.7 ± 3.7 % in AdIL-10-transduced MSCs; *p* = 0.0081) and inguinal (Fig. 3c; 31.6 ± 0.7 % in vehicle vs 26.6 ± 1.3 % in AdIL-10-transduced MSCs; *p* = 0.0238) lymph nodes as well as a decrease in the number of activated CD8^+^ T cells in the inguinal lymph nodes (Fig. 3d; 9.7 ± 1 % in hMSCs vs 4.7 ± 0.5 % in AdIL-10-transduced MSCs; *p* = 0.0131). Levels of T-cell-associated cytokines IFN-γ, IL-2 and IL-4 were undetectable in serum harvested from animals in all treatment groups at the experimental end point, as determined by multiplex ELISA (data not shown). Additionally, levels of CD11b^+^ and CD11b^+^ Ly6C hi cells in the popliteal lymph nodes (markers of pro-inflammatory monocytes) were similar in all pooled samples from each treatment group (Additional file 3: Figure S3A). However, a trend towards decreased levels of CD11b^+^ and CD11b^+^ Ly6C hi cells was observed in the inguinal lymph nodes following treatment with AdIL-10 virus alone. Levels of MCP-1 in serum harvested from animals at the experimental end point were similar between each treatment group (Additional file 3: Figure S3B).

### Injection of hMSCs does not prevent OA development or progression

**Fig. 4:**
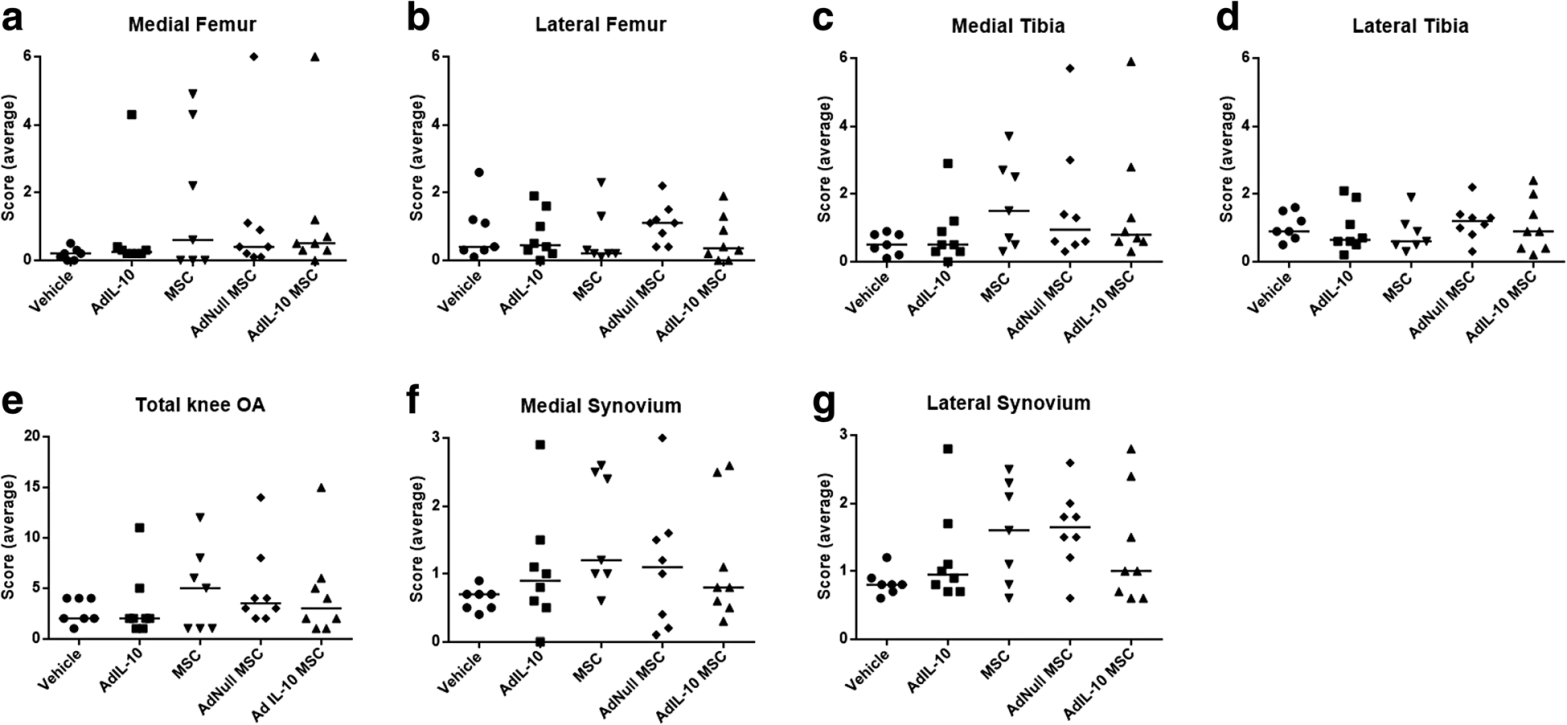
Injection of hMSCs does not prevent OA development or progression. Following two consecutive injections of 1 U collagenase over 2 days (1 U per day), mice were injected with untransduced, AdNull-transduced or AdIL-10-transduced hMSCs (or AdIL-10 virus or vehicle as control) at 1 week post OA induction. After 6 weeks knees were harvested and analysed for cartilage damage and synovial hyperplasia. **a**, **b** Median damage scores in the medial and lateral femur (maximum score of 6 with three blinded observers, approximately six sections per knee). **c**, **d** Median damage scores in the medial and lateral tibia compartments (maximum score of 6 with three blinded observers, approximately six sections per knee). **e** Total damage within the four joint compartments (maximum score of 24). **f**, **g** Median scores of synovial hyperplasia in the medial and lateral synovial compartments (maximum score of 3 with three blinded observers, approximately six sections per knee). Mild OA was observed in all groups with no significant reduction in the treated groups. Overall there appeared to be more damage in the medial compartment. *n* = 8 animals per group. *MSC* mesenchymal stem cell, *OA* osteoarthritis

**Fig. 5:**
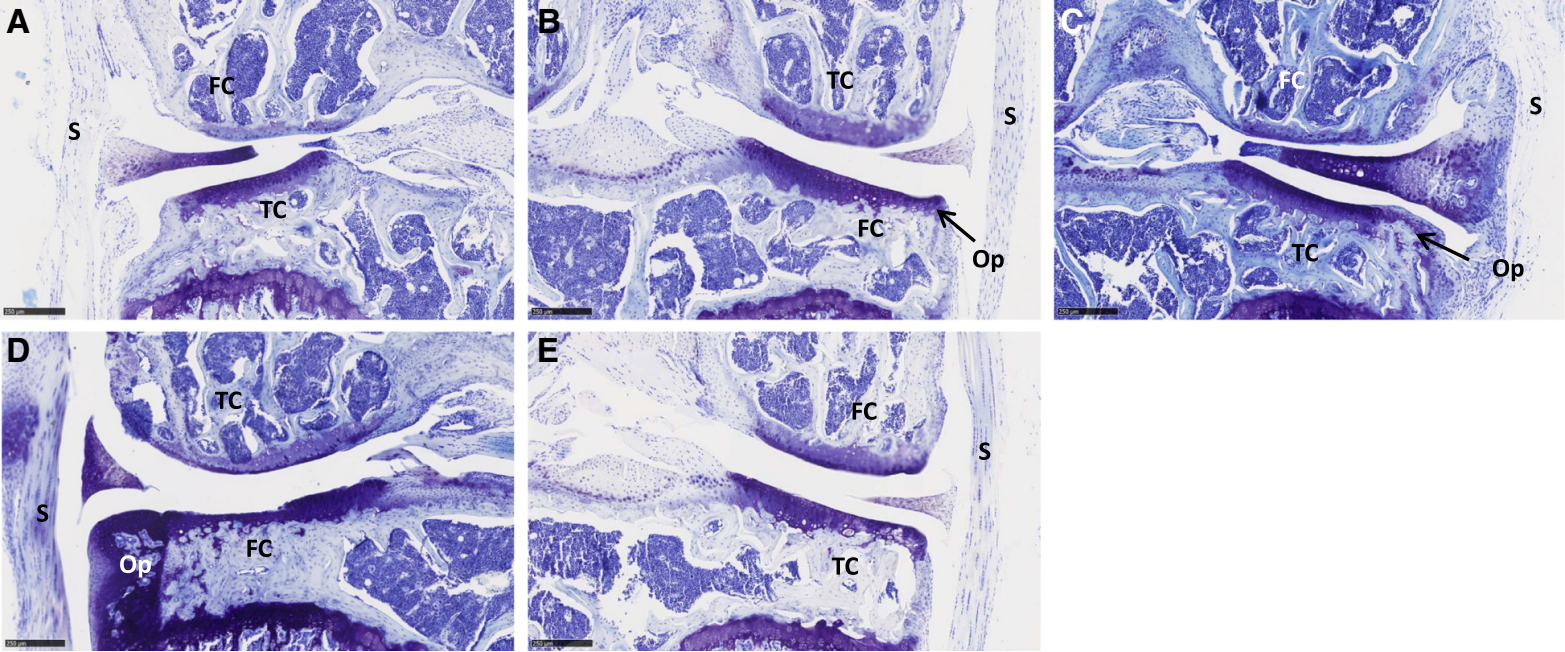
Representative images showing mild to severe OA. Representative thionine-stained sections of the median scoring knee from each condition: **a** vehicle, **b** Ad-IL10 only, **c** MSCs only, **d** AdNull MSCs and **e** Ad-IL10 MSCs. Erosion of the cartilage, osteophyte formation and synovial hyperplasia is visible in several of the images. Each image illustrates the medial compartment. *FC* femoral condyle, *TC* tibial condyle, *S* synovium, *Op* osteophyte. Scale bar =250 μm

To assess the effect of AdIL-10-transduced hMSCs on OA progression, knees treated with collagenase at time 0 and hMSCs at day 7 were harvested, sectioned and stained with thionine 6 weeks after injection of the cells. At this time point there was no significant difference observed between the amount of damage in vehicle-treated knees compared with any other group in any compartment (Fig. 4a–d). All knees showed changes to both the cartilage and synovium, with more damage observed in the medial compartment compared with the lateral. However, significant inter-animal variability was seen in groups injected with AdIL-10, MSCs, AdNull MSCs and AdIL-10 MSCs for cartilage and synovium changes in the medial compartment (Fig. 4a, c) and synovial hyperplasia in the lateral compartment (Fig. 4f). Histologically, joints with high scores presented with significant osteophyte formation and loss of medial compartment cartilage (Fig. 5b). This appeared to occur more in the hMSC-treated knees, irrespective of whether the cells were transduced or not. The same pattern was observed in the synovial damage scores, with more inflammation observed in the samples that received injections of hMSCs compared with vehicle (Fig 4f, g). Although not significant, joints treated with AdIL-10-transduced MSCs showed less synovial changes compared with the MSC-treated and AdNull MSC-treated joints. Ultimately, groups with the least damage were those that were treated with vehicle or with virus only (Fig. 4e).

## Discussion

Recently it has become clear that OA is a disease of the entire joint and not simply the cartilage [35]. Increasing evidence highlights the association between factors produced by inflamed synovium and the initiation and progression of the disease. Furthermore, inflammation of the synovial membrane with increased vascular density and cellular infiltration is a prominent feature of OA pathogenesis [36]. The aim of this research was to investigate the potential of vIL-10-overexpressing hMSCs to mitigate this inflammation, via modulation of the immune response and to assess whether this modulation could delay or prevent the OA onset and progression.

It is clear from the literature that T lymphocytes play a role in the pathogenesis of OA [37] with cellular infiltration of the synovial tissue leading to induction of inflammatory factors [4, 5, 38] and osteoclastogenesis [6]. Hsieh et al. [39] have shown that CD8 knockout animals exhibit reduced OA, and an increase in the presence of CD3^+^ cells has been demonstrated in the synovium of osteoarthritic animals [40]. Furthermore, many T-cell-associated factors, such as IL-17, have been shown to have negative effects upon joint cartilage [4, 41, 42]. While synovial inflammation is higher in rheumatoid arthritis compared with OA, osteoarthritic knees still have higher levels of inflammation compared with healthy controls [43]. Here we clearly demonstrate that the injection of vIL-10-overexpressing hMSCs leads to long-term reductions in the numbers of CD4^+^ and CD8^+^ T cells in the draining popliteal lymph nodes of the knees and activated CD4^+^ and CD8^+^ T cells in the inguinal lymph nodes. Also, injection of AdIL-10 virus alone led to similar outcomes in the levels of activated CD4^+^ cells in the popliteal lymph nodes. Furthermore, this was a long-lasting effect, with these reductions being observed 6 weeks after injection of hMSCs.

IL-10 is a pleiotropic cytokine that has been shown to have positive effects on the synthesis of collagen type II and aggrecan as well as reducing secretion of pro-inflammatory cytokines [44]. However, IL-10 has also been shown to induce proliferation and promote chondrogenic or hypertrophic differentiation of primary chondrocytes, highlighting a role for IL-10 in endochondral bone growth [45]. Elevated levels of IL-10 have been detected in cartilage, synovium and subchondral bone explants of patients with OA [46]. Despite the fact that IL-10 appears to play a protective role on cartilage in the joint, we did not observe a direct effect of vIL-10 overexpression on structural changes within the joint during development of OA. Further investigation is therefore required to evaluate the chondroprotective potential of vIL-10 and its ability to attenuate progression of OA. There is also the possibility that the presence of IL-10 in the osteoarthritic joint could lead to further cellular hypertrophy and a negative outcome since it is also associated with endochondral ossification [45]. However, no increased joint damage was seen in either vIL-10-treated group compared with the others. While it was not the focus of the study to understand subchondral bone changes, we cannot discount that there may have been differences in this region in vIL-10-treated animals. Furthermore, the effects of the inflammatory environment upon bone could be very different from those on cartilage. As such it is possible that this treatment, which had no clear effect on the joint cartilage, may have had subtle effects on the surrounding bone tissues.

MSCs are known to produce a host of paracrine factors that can reduce inflammation and induce tissue repair in a number of models [47]. However, whether they induce joint repair or simply reduce pain is not completely clear. It does appear that their mechanism of action is more related to paracrine effects and reduction of inflammation rather than by direct tissue generation/regeneration [48]. The exact efficacy of these MSCs in prevention or reversal is still unclear. While there have been some promising reports relating to the reduction of pain and even the repair of defects within the joint by MSCs, longer term and more comprehensive clinical trials are required in order to clearly identify the efficacy of MSC therapy. Schurgers et al. [49] previously reported differential effects of MSCs on T-cell proliferation in vitro vs in vivo using a collagen-induced arthritis (CIA) model. In contrast to these findings, we observed a decrease in T lymphocytes in vitro and in vivo in our OA model following treatment with hMSCs transduced to express vIL-10. However, even though we did not observe an immunogenic effect of untransduced or virally transduced hMSCs on mouse cells in vitro, we did observe that the majority of the severely damaged knees were in groups treated with the hMSCs or transduced hMSCs. Interestingly, fully genetically mismatched allogeneic MSCs have been shown to hold less immunosuppressive activity compared with a syngeneic or partially mismatched cell source, and have aggravated disease pathogenesis in a murine CIA model [50]. We hypothesise that our findings may be due to the xenogeneic nature of MSCs used in this study. It is possible that while there was a reduction in the levels of immune cells present and also in the TNF-α levels in vitro, vIL-10 overexpression was not sufficient to overcome these xenogeneic effects. Once again the effects on the bone density were not assessed and these effects should be taken into account for studies.

Our study has some limitations. Despite our observation of a long-term effect of vIL-10-overexpressing hMSCs on the T-cell number, we did not observe a large effect of this treatment on the severity and progression of OA. There are several possible reasons for this lack of effect on the severity and progression of OA. Firstly, the model used was designed to lead to mild OA. However, the degree of progression of the disease was slower/milder than expected at this time point based on pilot data (data not shown). Also there were large differences between animals in the levels of OA and joint damage. This led to a large amount of variation within and between groups. It is possible that a clearer picture might emerge with a slightly more severe OA model, larger animal numbers or a longer period between cell injection and joint harvest.

## Conclusions

We present a long-term immunomodulatory effect of vIL-10-overexpressing hMSCs injected into the knees of osteoarthritic mice. While there was no clear reduction in disease onset or progression, we believe that this approach could be used in combination with reparative approaches to result in a long-term reduction in joint inflammation. As stated above, T lymphocytes are the most abundant infiltrating immune cells present in OA synovium and a role for CD4^+^ T cells in the pathogenesis of OA has been elucidated (refs). Based on the reduction in activated T cells present in the draining lymph nodes of the knees, we believe that, in instances of OA with a high inflammatory component, treatment with a combination of syngeneic or perhaps allogeneic MSCs and vIL-10 could help to reduce symptoms and disease progression. We also feel that the results presented do somewhat call into question the use of xenogeneic cells for the investigation of MSC-mediated repair/protection of the joint following OA induction. This should be considered in the execution of similar experiments in the future.

## Additional files

Additional file 1: Figure S1. Timeline illustrating the induction of OA in mice followed by injection of five different treatment conditions at day 7. Six weeks after treatment animals were euthanised and the joints harvested for histological scoring. Lymph nodes and blood were taken for flow cytometric and multiplex ELISA analyses respectively. (PDF 9 kb) Additional file 2: Figure S2. Representative safranin O-stained sections of the median scoring knee from each condition as well as an untreated contralateral knee for comparison: **A** Vehicle, **B** Ad-IL10 only, **C** MSCs only, **D** AdNull MSCs, **E** Ad-IL10 MSCs and **F** untreated contralateral knee. Erosion of the cartilage, osteophyte formation and synovial hyperplasia is visible in several of the images. Each image illustrates the medial compartment. *FC* femoral condyle, *TC* tibial condyle, *S* synovium, *Op* osteophyte. Scale bar =250 μm. (PDF 197 kb) Additional file 3: Figure S3. AdIL-10-transduced MSCs do not reduce the levels of pro-inflammatory monocytes at 6 weeks post injection. **A** CD11b and Ly6C expression by myeloid cells isolated from the popliteal and inguinal lymph nodes at 6 weeks post treatment, as detected by flow cytometry. For the popliteal lymph nodes, data points represent *n* = 4, pooled from eight animals. Data points in the AdIL-10 group represent *n* = 5, with three samples pooled from two animals and two single samples. For inguinal lymph nodes, data points represent *n* = 2, pooled from eight animals. Data points in the AdIL-10 group represent *n* = 3, with one sample pooled from four animals, one sample pooled from three animals and one single sample. **B** Serum levels of MCP-1 at 6 weeks post treatment, as quantified utilising a chemiluminescent array. (PDF 47 kb)

## Abbreviations

α-MEM: alpha-Minimum Essential Medium
CFSE: carboxy-fluorescein diacetate, succinmidyl ester
CIA: collagen-induced arthritis
CM: conditioned medium
FBS: foetal bovine serum
hMSC: human mesenchymal stem cell
IL: interleukin
LPS: lipopolysaccharide
MCP-1: monocyte chemoattractant protein-1
MHC: major histocompatibility complex class II
MSC: mesenchymal stem cell
OA: osteoarthritis
TNF-α: tumour necrosis factor alpha
vIL-10: viral interleukin-10
