# Supplementary material for: vIL-10-overexpressing human MSCs modulate naïve and activated T lymphocytes following induction of collagenase-induced osteoarthritis
Source: Stem Cell Res Ther. 2016 May 18;7:74. doi: 10.1186/s13287-016-0331-2 (PMC4870800; doi:10.1186/s13287-016-0331-2)
Supplement: Additional file 1: Figure S1. — Timeline illustrating the induction of OA in mice followed by injection of five different treatment conditions at day 7. Six weeks after treatment animals were euthanised and the joints harvested for histological scoring. Lymph nodes and blood were taken for flow cytometric and multiplex ELISA analyses respectively. (PDF 9 kb) [file 13287_2016_331_MOESM1_ESM.pdf]

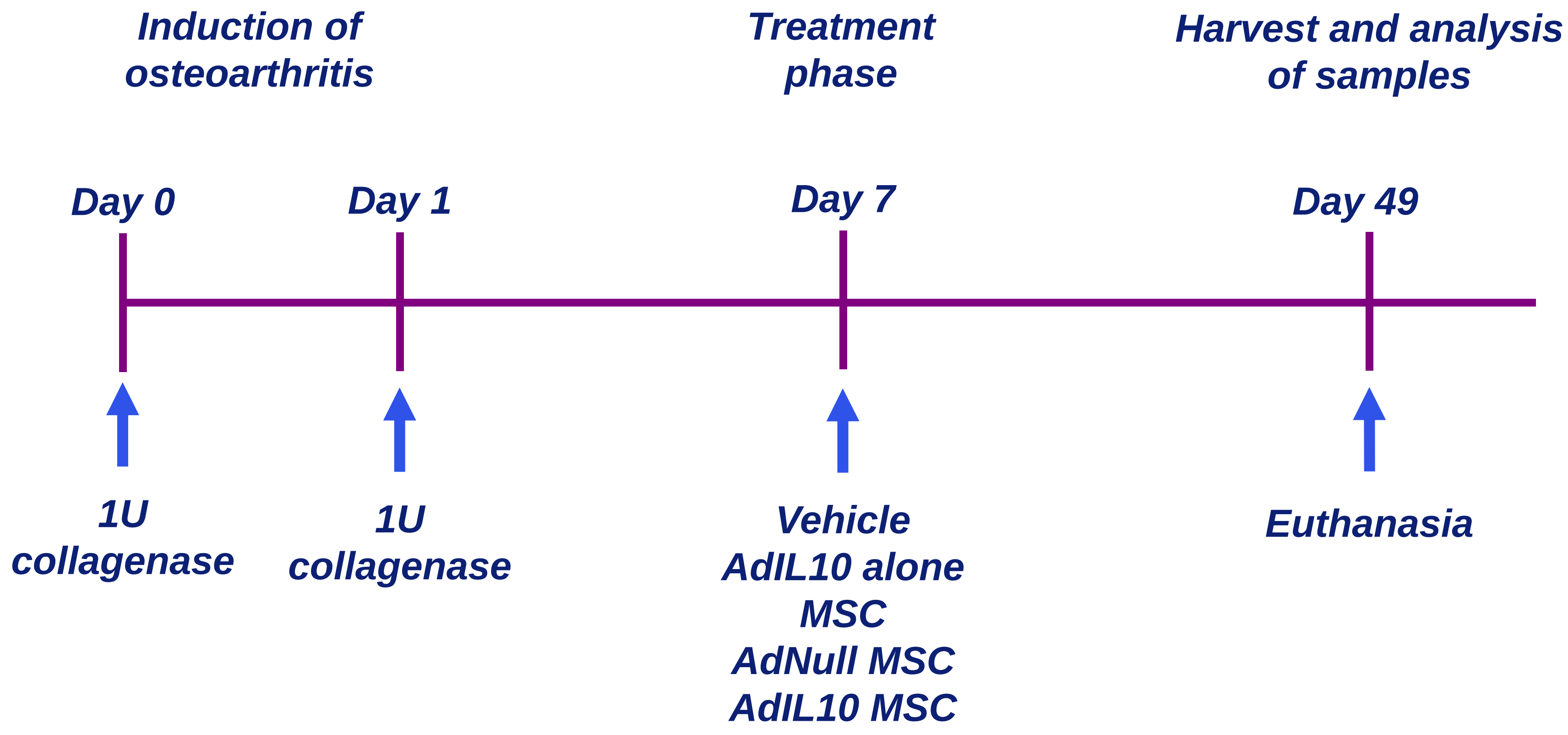

Induction of osteoarthritis
Treatment phase
Harvest and analysis of samples
Day 0
Day 1
Day 7
Day 49
1U collagenase
1U collagenase
Vehicle
AdIL10 alone
MSC
AdNull MSC
AdIL10 MSC
Euthanasia
