# Supplementary figures and images for: vIL-10-overexpressing human MSCs modulate naïve and activated T lymphocytes following induction of collagenase-induced osteoarthritis
Source: Stem Cell Res Ther. 2016 May 18;7:74. doi: 10.1186/s13287-016-0331-2 (PMC4870800; doi:10.1186/s13287-016-0331-2)

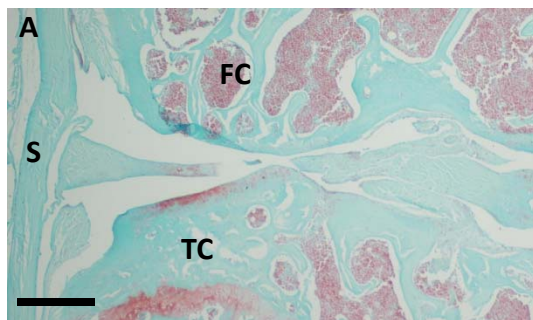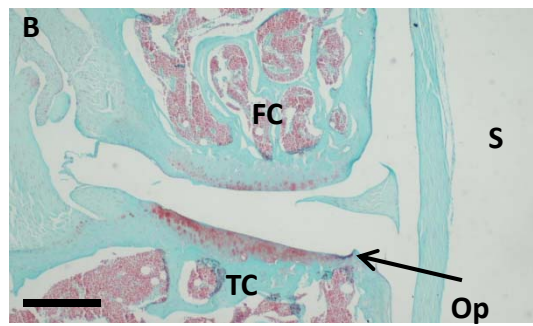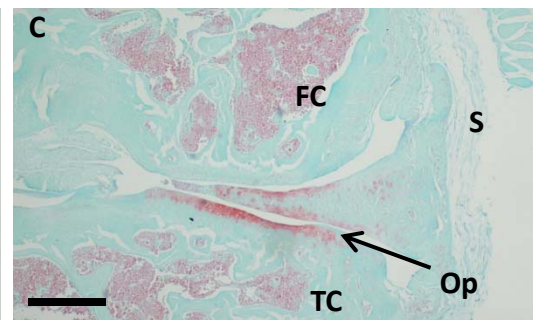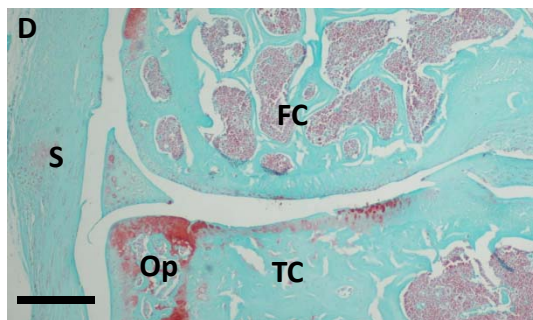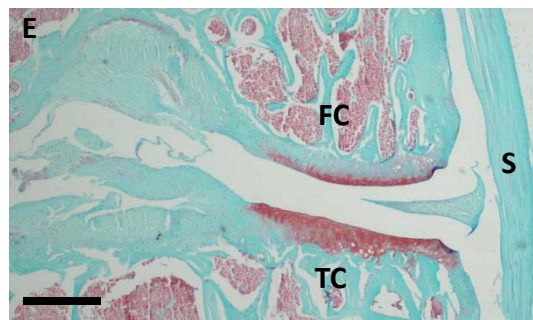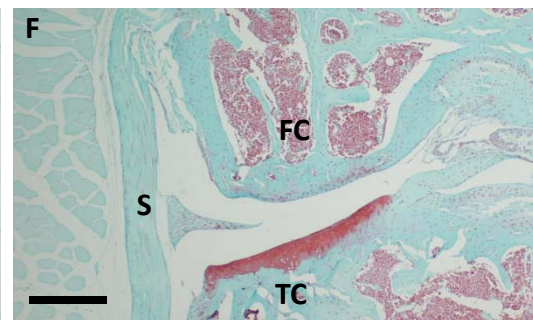

Supplement: Additional file 2: Figure S2. — Representative safranin O-stained sections of the median scoring knee from each condition as well as an untreated contralateral knee for comparison: A Vehicle, B Ad-IL10 only, C MSCs only, D AdNull MSCs, E Ad-IL10 MSCs and F untreated contralateral knee. Erosion of the cartilage, osteophyte formation and synovial hyperplasia is visible in several of the images. Each image illustrates the medial compartment. FC femoral condyle, TC tibial condyle, S synovium, Op osteophyte. Scale bar =250 μm. (PDF 197 kb) [file 13287_2016_331_MOESM2_ESM.pdf]

**A**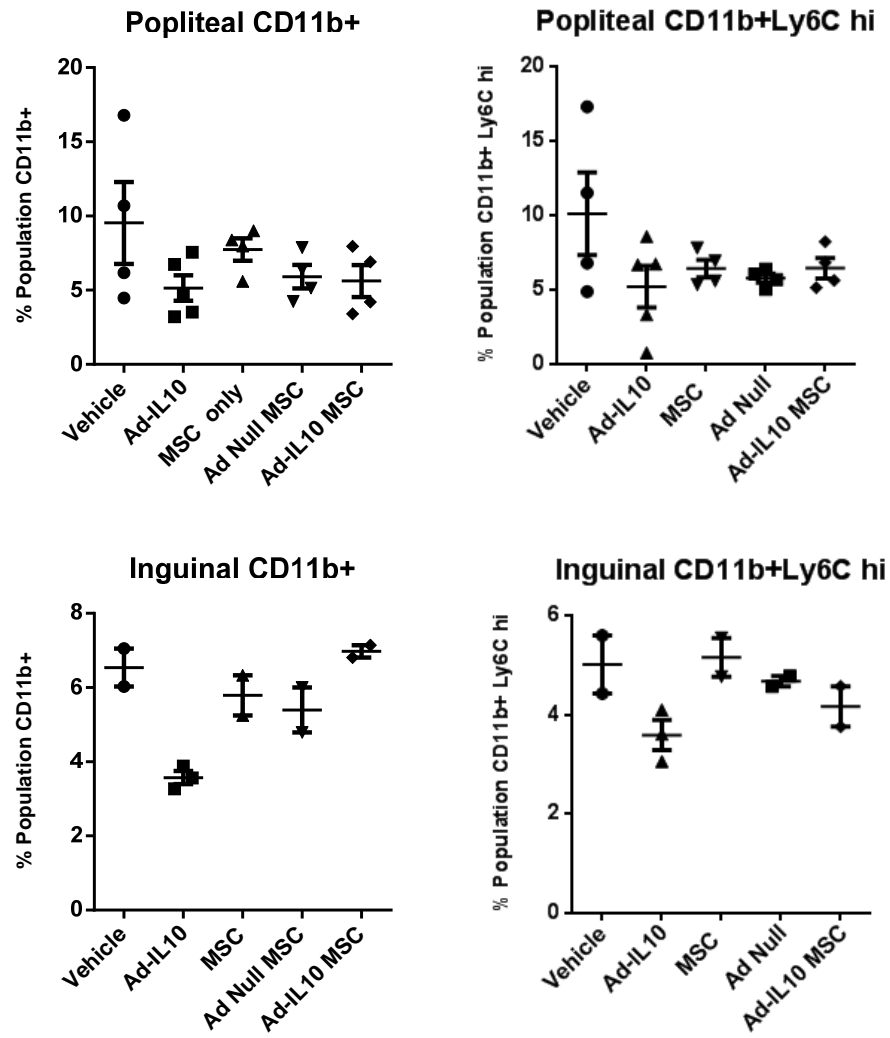**B**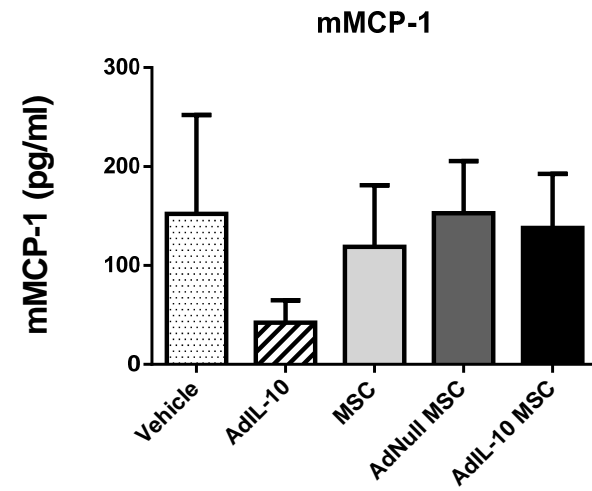

Supplement: Additional file 3: Figure S3. — AdIL-10-transduced MSCs do not reduce the levels of pro-inflammatory monocytes at 6 weeks post injection. A CD11b and Ly6C expression by myeloid cells isolated from the popliteal and inguinal lymph nodes at 6 weeks post treatment, as detected by flow cytometry. For the popliteal lymph nodes, data points represent n = 4, pooled from eight animals. Data points in the AdIL-10 group represent n = 5, with three samples pooled from two animals and two single samples. For inguinal lymph nodes, data points represent n = 2, pooled from eight animals. Data points in the AdIL-10 group represent n = 3, with one sample pooled from four animals, one sample pooled from three animals and one single sample. B Serum levels of MCP-1 at 6 weeks post treatment, as quantified utilising a chemiluminescent array. (PDF 47 kb) [file 13287_2016_331_MOESM3_ESM.pdf]
